# Evidence of Reduced Global Processing in Autism Spectrum Disorder

**DOI:** 10.1007/s10803-016-2724-6

**Published:** 2016-02-10

**Authors:** Rhonda D. L. Booth, Francesca G. E. Happé

**Affiliations:** 1 Cognitive Neuroscience and Neuropsychiatry Section, Institute of Child Health, University College London, 30 Guilford Street, London, WC1N 1EH UK; 2 MRC Social, Genetic & Developmental Psychiatry Centre, Institute of Psychiatry, Psychology and Neuroscience, King’s College London, Denmark Hill, London, SE5 8AF UK

**Keywords:** Local–global processing, Weak central coherence (wCC), ASD, Impossible-Figures, Fragmented Picture-Completion

## Abstract

Frith’s original notion of ‘weak central coherence’ suggested that increased local processing in autism spectrum disorder (ASD) resulted from reduced global processing. More recent accounts have emphasised superior local perception and suggested intact global integration. However, tasks often place local and global processing in direct trade-off, making it difficult to determine whether group differences reflect reduced global processing, increased local processing, or both. We present two measures of global integration in which poor performance could not reflect increased local processing. ASD participants were slower to identify fragmented figures and less sensitive to global geometric impossibility than IQ-matched controls. These findings suggest that reduced global integration comprises one important facet of weak central coherence in ASD.

## Introduction

Frith was perhaps the first to focus on assets and superior performance in those with autism spectrum disorder (ASD) as being more informative than task failure (Frith 1989; Shah and Frith 1983, 1993). Her notion of ‘weak central coherence’ (wCC) explained superior processing on tasks in which local bias is advantageous (e.g., Embedded Figures Test, EFT; Witkin et al. 1971; Block Design subtest from the Wechsler scales; Wechsler 1992), as due to reduced pull of global form/gestalt and an unusual ability to disregard context with its camouflaging effect on perception of local features.

Since her original formulation, the notion of detail-focused processing in ASD has attracted a great deal of research and several alternative theoretical accounts (for review, see Happé and Booth 2008; Happé and Frith 2006; Van der Hallen et al. 2015). In particular, alternative theories such as Enhanced Perceptual Functioning (Mottron and Burack 2001; Mottron et al. 2006) and superior ‘Systemising’ in the ‘extreme male brain’ (Baron-Cohen 2002) have suggested that local processing is superior in ASD alongside intact global processing. A number of studies have reported a featural processing bias and unimpaired global processing ability in ASD. For example, Hadad and Ziv (2015) argued that although their participants with ASD demonstrated a bias towards analytic perception, they were still sensitive to effects of Gestalt grouping laws (but see, e.g., Brosnan et al. 2004 for evidence of reduced gestalt grouping in ASD). Almeida et al. (2014) also found superior contour integration abilities in individuals with high levels of autistic traits, alongside enhanced local processing skills (EFT). Other studies, such as that by Koldewyn et al. (2013), suggest intact global processing but a bias towards local processing in tasks tapping preferred processing style rather than (directed) task ability.

Traditional paradigms used to measure coherence tend to conflate global and local processing—often placing them in trade-off. In such cases it is difficult to determine whether patterns of performance in autism reflect reduced global processing, increased local processing, or both. For example, superior performance on the EFT in ASD could result from either superior processing of the local form or a reduced camouflaging effect of the global gestalt. The aim of the present study was to assess global integration of visual information in ASD with two tasks selected to minimise the trade-off between global and local processing. The two measures were (1) a modified Fragmented Picture-Completion Task that required participants to identify a picture from fragments; and (2) an Im/possible Figures Task, requiring judgement of “possible” or “impossible” figures. Each task required integration of global visual information, predicted to be reduced in ASD. Group differences on the tasks were unlikely to reflect increased local processing in ASD, as described below.

### Fragmented Picture-Completion Task

Gestalt completion or perceptual closure tasks (e.g., the Gestalt Closure subtest from the Kaufman Assessment Battery for Children; Kaufman and Kaufman 1983), require the participant to identify partially completed drawings of common objects. Success relies on strong visual coherence in order to “combine disconnected, vague, visual stimuli into a meaningful whole” (Carroll 1993, p. 308).

**Fig. 1:**
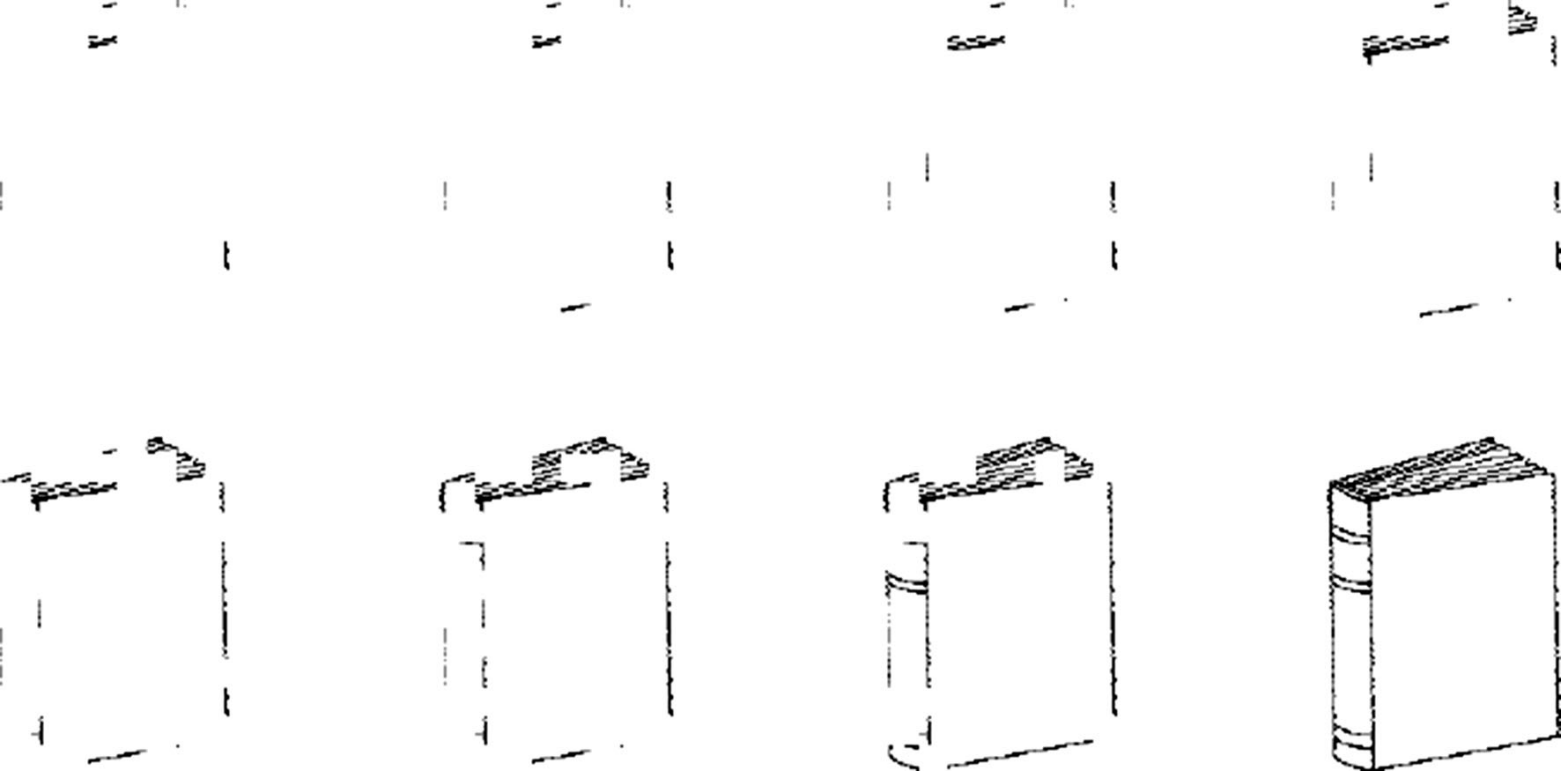
Example of consecutive frames from the Fragmented Picture-Completion Task (reproduced by kind permission, Snodgrass et al. 1987)

Snodgrass et al. (1987) developed the Fragmented Picture-Completion Task (based on the Gollin Figures; Gollin 1960). Line drawings of common objects, taken from the Snodgrass and Vanderwart (1980) picture set, were fragmented by the random deletion of pixels into a series of eight images (see Fig. 1 for an example). The most fragmented level is presented on a computer screen and participants advance through each successive image until they recognise the complete form. Although the Fragmented Picture-Completion Task was devised for experiments on implicit memory, a wealth of normative data exists on identification thresholds for 150 picture stimuli (Koch et al. 1995; Snodgrass and Corwin 1988; Wyatt et al. 1998). The present study used the images developed by Snodgrass et al. in an adaptation of their Fragmented Picture-Completion Task.

Previous work on visual integration in ASD has yielded mixed results. Jolliffe and Baron-Cohen (2001) reported that individuals with ASD were less able to integrate visual elements using a modified version of the Hooper Visual Organisation Test (Hooper 1983) where objects have to be identified from parts positioned randomly on a page. Interestingly, Jolliffe and Baron-Cohen found participants with ASD showed no impairment in object identification from a single piece, but did show a deficit in the ability to conceptually integrate single elements in order to form a meaningful whole (when potentially identifying single details were absent). Mottron and Belleville (1993) assessed the visual integration capabilities of their single case of a savant artist with Asperger syndrome, and found no differences compared to control participants in the ability to recognise degraded pictures using Gollin’s graded picture series or the Hooper Visual Organisation Test. Scheurich et al. (2010) reported significantly lower recognition of fragmented pictures in ASD compared to control participants, although differences did not hold after controlling for age and nonverbal intelligence. Most recently, Evers et al. (2014) tested children and adolescents with ASD on a contour identification task using Gaborized object outlines, and found an identification disadvantage compared to age and IQ-matched typically-developing (TD) participants. Similarly, Olu-Lafe et al. (2014) found their sample of adults and adolescents with ASD were significantly slower to mentally integrate two complex shapes into a single figure compared to age and IQ-matched controls.

### Im/possible Figures Task

**Fig. 2:**
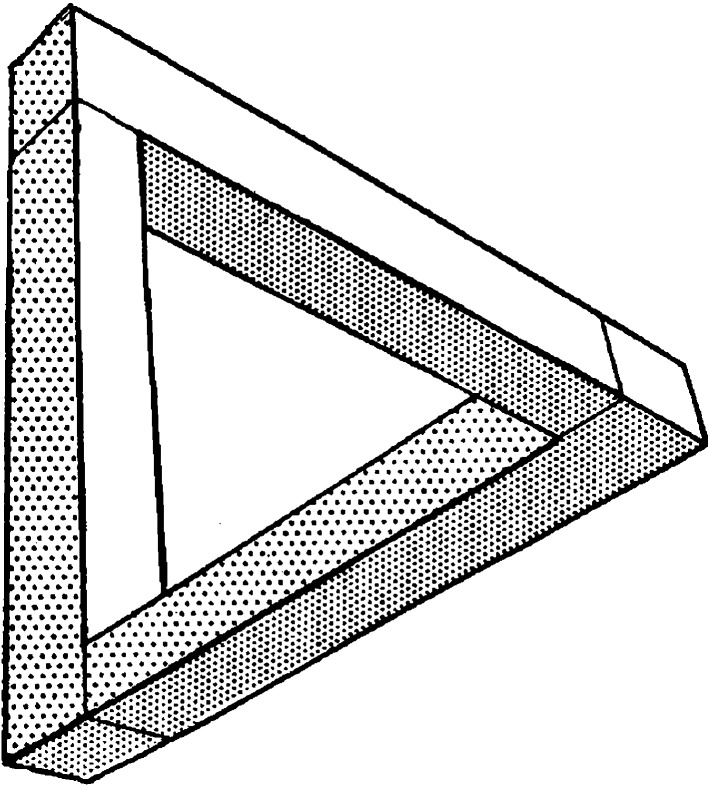
An impossible triangle (Penrose and Penrose 1958)

Impossible figures are drawings of geometric forms that would be impossible to construct in three-dimensions. A classic example is the Penrose triangle, first described in Penrose and Penrose (1958) (see Fig. 2), which is geometrically possible at each corner, but presents a contradiction when viewed as a whole. The figure is therefore locally possible but globally impossible as a unified three-dimensional object. Impossibility is said to be an emergent property of the whole figure, and requires the global integration of local parts in order to be detected (Young and Deregowski 1981). As visual coherence is required to identify impossibility, an impossible figures detection task was used as a measure of configural processing in the present study.

Research suggests individuals with ASD do not readily perceive the impossibility of impossible figures. Mottron and Belleville (1993) reported impairment in the perception of impossible figures (at brief durations) in their case study of EC, a 34-year-old male with Asperger syndrome and exceptional graphic drawing skills. When asked to draw impossible figures from memory, E.C. would produce globally coherent figures that did not include the impossible element.

Mottron et al. (1999) explored the perception of impossibility in 10 high-functioning adults and adolescents with autism and 10 age- and IQ-matched controls. They found that both groups took longer to copy impossible figures compared to their possible counterparts, even though the figures were matched for number and type of features. The difference between conditions was significantly reduced in the autism group however, suggesting that these individuals were not as affected by geometric impossibility.

Rodgers (2000) also examined the ability to detect geometric impossibility in her study of eight adults with Asperger syndrome. Pairs of matching possible and impossible figures were presented simultaneously; individuals with Asperger syndrome made significantly more errors in identifying which figure was impossible, compared to an age- and IQ-matched control group.

Both the Fragmented Picture-Completion Task and the Impossible Figures Task were included in the present study because they appear to tap global visual integration relatively unconfounded by local processing bias. Poor performance would be predicted in ASD if global integration is reduced, but superior local processing alone (as predicted by Enhanced Perceptual Functioning and Systemising theories) would not lead to poorer performance in ASD versus TD groups.

### Hypotheses

The original weak coherence account of ASD predicts that individuals with ASD will be less proficient at integrating featural information. By contrast, the Enhanced Perceptual Functioning and Systemising accounts would predict no group difference on a task where global processing is required and local processing superiority is neither advantageous nor disadvantageous. We therefore tested two hypotheses from wCC; that individuals with ASD would: (1) show a relative inferiority on a Fragmented Picture-Completion Task, requiring more fragments of the image to be displayed/cohered and more processing time for correct identification, relative to age- and IQ-matched controls; (2) be less proficient at discriminating possible from impossible geometric figures compared to age- and IQ-matched controls as shown by more errors and slower responses. Furthermore, if both tasks measure global integration, performance across tasks would be predicted to correlate.

## Methods

### Participants

The ASD group comprised 26 males (9–21 years of age, Full-Scale IQ: FIQ range = 49–134) with a formal diagnosis of an ASD; autism (n = 6) or Asperger syndrome (n = 20). All ASD participants had been diagnosed independently by a qualified clinician (psychiatrist or clinical psychologist) using DSM-IV (Diagnostic and Statistical Manual of Mental Disorders) criteria (American Psychiatric Association 1994). Admission to the specialist educational placements from which the participants were recruited required a formal diagnosis of autism/Asperger syndrome. Any individual for whom detailed information about source of diagnosis was lacking was excluded from the study. Furthermore, due to the attentional demands of the tasks, participants were excluded if they had comorbid Attention Deficit Hyperactivity Disorder (ADHD), attention deficit disorder (ADD), hyperkinetic disorder, and/or Tourette syndrome.

Participants were recruited from two residential schools (one specializing in Asperger syndrome and one for children with a range of special educational needs) and parent group contacts. Current FIQ data (measured within 4 years) from the Wechsler Intelligence Scale for Children (WISC-III; Wechsler 1992) or Wechsler Adult Intelligence Scale (WAIS-III; Wechsler 1997) were available or collected by the experimenter for 11 participants in the ASD group. Due to time constraints, 15 participants were administered a short form Wechsler IQ assessment to obtain FIQ, VIQ, and PIQ estimates. This was based on four subtests that have been reported to have high reliability (Sattler 1992): Information and Vocabulary (for VIQ), and Picture Completion and Block Design (for PIQ), combined for FIQ. The use of short forms to estimate IQ in ASD populations has been validated by Minshew et al. (2005).

The control group comprised 30 males individually matched in age (range = 9–20 years) and ability (FIQ range = 47–140 using the short form described above) to participants in the ASD group. Of these, four participants with moderate learning disability (MLD, the term used for intellectual impairment in the United Kingdom) were recruited from a special educational needs school to match low-functioning participants in the ASD group. The remaining control participants were selected from a large study examining individual differences in processing style in typical development (Booth 2006). School-aged participants were recruited from three secondary schools and two primary schools. Adult participants were recruited through advertisements placed in job centres, public libraries, youth clubs, hospital notice boards, and shop windows. Participants were required to have English as a first language, no clinically significant impairment or diagnosis, and no family history of ASD. Participants spanned a wide range of ethnic backgrounds and socioeconomic status (SES), but the majority were of White British origin and average SES for southern Great Britain.

Participants were excluded from the control group if they had fragile X syndrome or any suggestion of an ASD. As a screening measure, parents of MLD children completed the Social Communication Questionnaire (SCQ; Berument et al. 1999), a brief checklist derived from the Autism Diagnostic Interview (ADI-R) algorithm items (Lord et al. 1994). Participants were excluded from the study if their SCQ score fell in the ASD range in social, communication and rigid/repetitive domains.

Participant characteristics for the ASD and control groups are presented in Table 1. Statistical comparisons confirmed that the ASD and control groups did not differ significantly in age or IQ.

**Table 1:** Participant characteristics by group: *M (SD)*

	ASD (*N* = 26)	Control (*N* = 30)	*t*	*p*	Cohen’s *δ*
Age	14.7 (2.3)	14.9 (2.4)	0.28	.78	0.09
FIQ	91.9 (24.7)	95.6 (19.7)	0.62	.54	0.17
VIQ	95.3 (24.4)	98.7 (20.4)	0.57	.57	0.15
PIQ	89.7 (22.2)	93.2 (17.5)	0.67	.51	0.18

### Materials

#### Fragmented Picture-Completion Task

Ten picture sequences were selected from the Fragmented Picture-Completion Task (Snodgrass et al. 1987): apple, elephant, pig, sock, television, kite, snowman, cake, book, and pear. The difficulty level of these items was “moderate” according to the norms collected by Snodgrass and Corwin (1988); that is, they were correctly identified by 35 percent of adults by the fourth frame (from a maximum of eight). Items of moderate difficulty were considered to be more applicable for participants with a range of ability levels, especially those with suspected global processing deficits. An example of consecutive frames for a stimulus picture is shown in Fig. 1.

It was checked that pictures could not be identified on the basis of individual parts alone, such that critical features (e.g., an eye or a tail in a picture of an animal) were not present at the most fragmented levels (i.e., from the first to the fourth frame). This procedure was adopted by Jolliffe and Baron-Cohen (2001) in their modification of the Hooper Visual Organisation Test, and ensures that the task assesses visual integration ability, rather than successful recognition based on an isolated element. Pictures were also selected on the basis of high name agreement and familiarity as rated by young children (Cycowicz et al. 1997). The items were piloted by the first author on a sample of TD children (N = 44, age range = 7–15 years) to ensure that all items could be named at the final frame, and that performance showed good inter-participant variability, without ceiling or floor effects.

#### Im/possible Figures Task

**Fig. 3:**
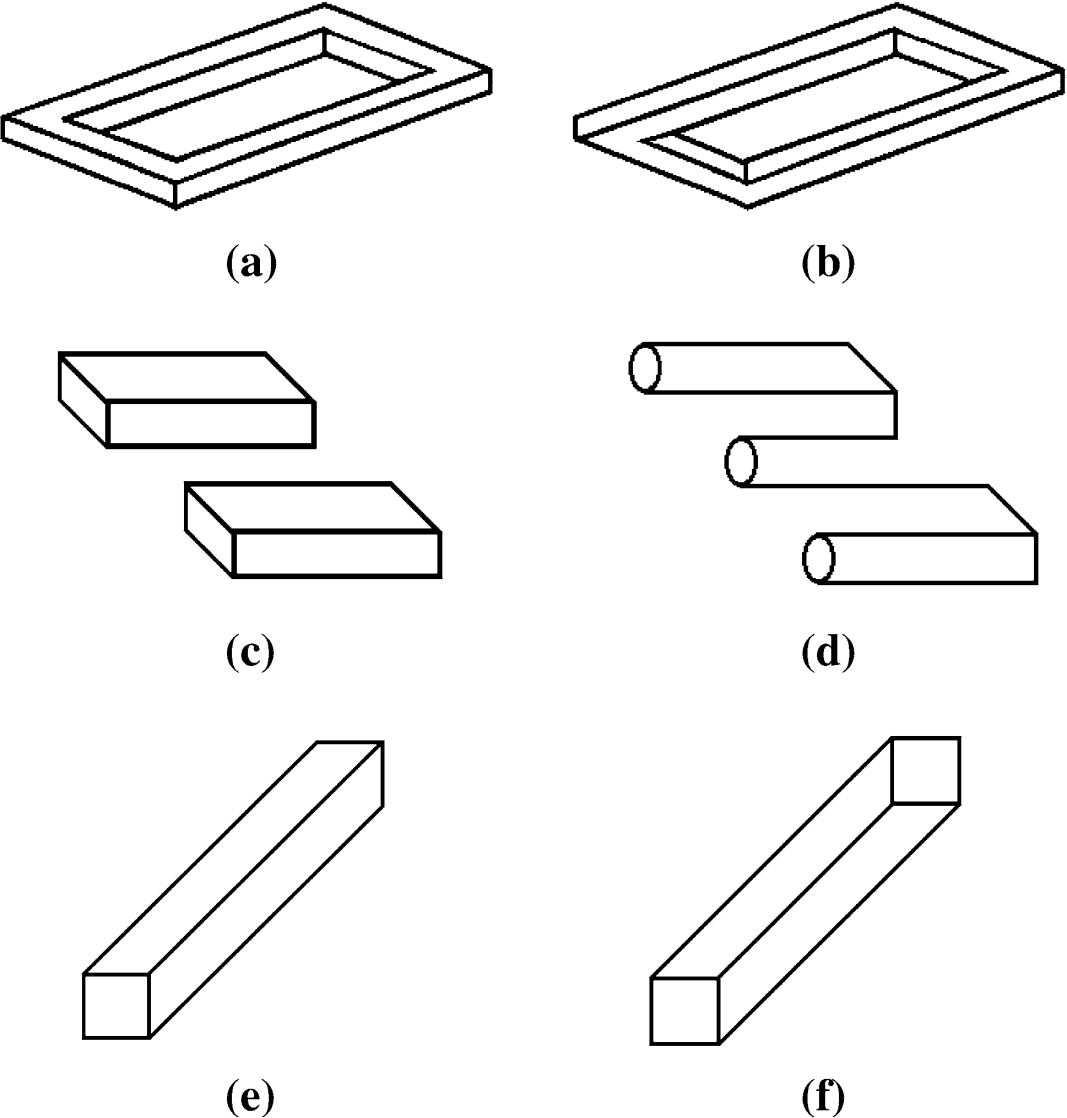
Examples of stimuli from the Im/possible Figures Task: **a** possible and **b** impossible forms of the rectangle; **c** possible and **d** impossible forms of the simple trident; **e** possible and **f** impossible forms the rod

Test stimuli for the Im/possible Figures Task consisted of 16 geometric Figures (8 possible and 8 impossible figure versions, in matched pairs), adapted from Young and Deregowski (1981), Robinson and Wilson (1973) and Terouanne (1980). An example pair is shown in Fig. 3a, b, and the full set of stimuli can be seen in the Appendix. A larger set of 20 figures was piloted with 32 TD individuals aged 8–15 years, during which appropriate introduction of the task and concept of ‘impossibility’ was developed. Item analysis conducted on data from a large TD sample (N = 204) suggested that two figures (triangle, pentagon) were difficult to judge for impossibility (correct detection rates < 70 %), and these were removed.

Both the modified Fragmented Picture-Completion Task and the Im/possible Figures Task were presented using SuperLab Pro software controlled by a laptop computer. Pictures appeared within a 3.25 by 3.25 inch (8.26 by 8.26 cm) square, positioned centrally, on a 15-inch computer touch screen. At a typical viewing distance of 50 cm, the stimuli subtended approximately 5.7°–10.2° of visual angle.

### Procedure

Ethical approval was obtained from the local research ethics committee of the Institute of Psychiatry at King’s College London (Study No. 034/99). Informed written consent was obtained from a parent or guardian for every school-aged participant, whereas those who had left school gave their own written consent to take part. Testing took place within the context of a larger study that consisted of three sessions lasting approximately 1 h (see Booth 2006; Booth and Happé 2010 for results from other parts of the battery). Both the Fragmented Picture-Completion Task and the Im/possible Figures Task were administered in the third session (approximately 30 and 50 min from the start of the session respectively) and were interspersed by a variety of visuospatial and verbal tasks in both computer and pencil-and-paper formats. All participants were tested individually in a quiet room with minimal distractions.

#### Fragmented Picture-Completion Task

To introduce the concept of fragmentation a complete picture of a chair first appeared on the computer screen. Participants were told to watch the picture as it was slowly going to disappear. The image was successively replaced every 5 s by a less complete image and the participant was encouraged to say when they could no longer recognise the picture as a chair. It was then explained that on this task they would see the opposite; pictures of objects were slowly going to appear on the screen. Their task was to watch the screen and tell the researcher as soon as they could recognise the picture.

Each fragmented image was presented one at a time for five-second exposures, from the most fragmented image (first frame) through to the complete image (eighth frame). When the participant gave a response the researcher immediately suspended the program (including the timer). The researcher informed the participant of the correctness of their response. If they were incorrect, they were told to keep looking and the program (including the timer) restarted from the beginning of the frame at which it was suspended. If they were correct, the researcher congratulated the participant and moved the program on manually until the complete image appeared. The researcher then began a new trial starting with the most fragmented image of the next object.

A rating scale was also included to check for possible group differences in willingness to guess the figure identity. If the ASD group had been more reluctant to guess, this might have appeared as slow recognition of the figures. The experimenter therefore asked participants to indicate how certain they were of their answers on a 3-point rating scale from “not that sure” to “very sure” before informing the participant whether they were correct or incorrect. As no group differences were found in how individuals judged the accuracy of their response, details of the certainty ratings are not presented here but can be obtained from the first author.

For each participant the following indices of task performance were taken: (1) the mean frame number (ranging from one to eight) at which the item was correctly identified; (2) the total number of incorrect responses (i.e., guesses); (3) the response time for when the item was correctly identified (since time was recorded from the presentation of the most fragmented image until the participant provided a correct response this index included a summation of times when incorrect responses were given).

#### Im/possible Figures Task

The Im/possible Figures Task began with an introduction phase to ensure that participants understood the concept of geometrical possibility. Possible and impossible forms of the simple trident were presented together on the touch screen (see Fig. 3c, d). The researcher told the participant that one of the two objects was real and could be made out of wood, but the other could not because there was something wrong with the drawing. The participant was then asked to select which object was ‘real’ and ‘possible’. If the participant touched the possible trident a congratulatory sound was played (Windows sound file: utopia asterisk.wav). If the participant touched the impossible trident the researcher explained how the figure was not possible and encouraged the participant to touch the possible figure. Once the correct selection was made a second example appeared consisting of the possible and impossible forms of a rod (see Fig. 3e, f). The participant was again encouraged to select the possible figure and the program moved on only after the correct selection was made.

Four practice trials were administered following the introduction. Participants were told figures would appear one at a time, and they had to decide whether each one was possible or impossible. Participants indicated their answer by touching the word *possible* or *impossible* presented at the bottom left and right of the screen respectively (or saying the word if preferred for lower-functioning participants). The four stimuli from the introduction phase were used in the practice trials. Corrective feedback was provided during the practice trials but not during the test phase.

When it was established that the participant understood the task requirements, the test phase began. Participants were reminded that they were being timed by the computer so to make their decision quickly, but as accurately as possible. The set of eight possible and impossible figures was presented in a fixed random order. Each figure remained on the screen until the participant made a response. Accuracy and time to respond from the onset of each figure were recorded.

## Results

### Fragmented Picture-Completion Task

Table 2 presents descriptive statistics for the Fragmented Picture-Completion Task, split by group. As our a priori predictions were directional, one-tailed tests were used for the key indices (frame number and response time for correct detection), with a significance level of *p* < .05. As we had no predictions on the direction of performance for the number of incorrect responses on the Fragmented Picture-Completion Task, two-tailed tests were applied.

**Table 2:** Global integration task results by group: *M (SD)*

	ASD (*N* = 26)	Control (*N* = 30)	*t*	*p*	Cohen’s *δ*
Fragmented Picture-Completion Task					
Frame number for correct detection (max = 8)	5.35 (0.50)	5.14 (0.45)	1.64	.053^c^	0.45
Total number of incorrect responses	2.73 (2.68)	1.67 (1.97)	1.71	.09	0.46
Response time for correct detection (s)	24.9 (2.6)	23.4 (2.4)	2.31	.01^c^	0.61
Im/possible Figures Task^a^					
Sensitivity A′	0.85 (0.15)	0.90 (0.12)	1.65^b^	.049^c^	0.23^d^
Response bias B″	0.28 (0.67)	0.29 (0.63)	0.01^b^	.99	0.001^d^
Possible figures proportion of correct detections	0.84 (0.19)	0.91 (0.13)	1.32^b^	.09^c^	0.18^d^
Impossible figures proportion of correct detections	0.74 (0.21)	0.80 (0.22)	1.48^b^	.07^c^	0.20^d^
Response time for possible figures (s)	1.87 (0.60)	1.97 (0.85)	0.05^b^	.48^c^	0.01^d^
Response time for impossible figures (s)	2.35 (0.84)	2.36 (1.02)	0.25^b^	.40^c^	0.03^d^

^a^ASD *n* = 25, control *n* = 28

^b^Mann–Whitney *U* tests, *z*-scores

^c^One-tailed tests

^d^Nonparametric effect size index $\gamma = \frac{Z}{\sqrt N }$

Accuracy rates were very high for both groups and only two ASD participants (aged 9 and 16 years) failed to name a picture in its complete form (pear, television). There were no significant group differences on the number of incorrect responses, indicating that the ASD group did not show any hesitancy in providing guesses.

Independent t-tests (one-tailed) showed a significant group difference on the mean response time for correct detection with the ASD group identifying the object significantly later than the control group. A trend towards significance was found on the mean frame for correct detection, with the ASD group identifying the object on average at a later frame. As the ASD group were slower to identify fragmented figures and required more detail before correct recognition (with medium effect sizes of .61 and .45 respectively) this suggests a difficulty in cohering fragmented information.

A qualitative analysis of the nature of incorrect responses was conducted. There was no indication of ‘isolate’ responses (Hooper 1983) from either group; that is, few incorrect responses could be interpreted as being based on a local detail. Instead most errors suggested more misinterpretation of global forms; for example “bottle”, “balloon”, or “lightbulb” for the pear stimulus.

A strong association between FIQ and visual integration ability was found in the ASD group with high FIQ relating to the identification of objects at an earlier level of fragmentation (Pearson product-moment correlation *r* = −.54, *p* = .005). In contrast, no relationship between FIQ and task performance was found in the control group (*r* = −.09, *p* = .64). Furthermore, the magnitude of the correlation coefficients between task performance and FIQ were significantly higher in the ASD group than the control group (Fisher r-to-z transformation; *z*_r1–r2_ = 1.81, *p* = .04). No association was found between age and task performance in either group (both *r* < −.26, *p* > .17).

### Im/possible Figures Task

All participants demonstrated an understanding of geometric impossibility in the introduction and practice trials of the Im/possible Figures Task; however three participants (one ASD, two controls) subsequently performed below chance on the test stimuli, suggesting they had not fully understood the task. Their data were therefore removed from the analyses. Statistical comparisons confirmed that the revised ASD and control groups did not differ significantly in age or IQ (all *t* < 0.85, *p* > .40).

As participants had an unlimited time to respond to each figure, response times for individual items were inspected for outliers. No time data were below 250 ms, while on four occasions response time exceeded 10 s (three occasions for impossible figures, one occasion for a possible figure). Two extreme times were from one ASD participant (12–27 s), while the remaining times came from control participants (11–12 s). All response times were consequently capped to 10 s.

Table 2 presents the mean proportion of correctly judged possible and impossible figures and the corresponding nonparametric indices of sensitivity^1^ A′ (combining correct detections of impossible figures and incorrect detections of possible figures) and response bias^2^ B″ for each group. Mean response times in detecting possible and impossible figures are also reported. Analyses were also conducted on correct response times but as the pattern of results did not change, all times are reported. As the data were strongly negatively skewed for all indices (*z*-scores ranged from −2.2 to −4.0) nonparametric analyses were used. One-tailed tests were applied to key indices where a priori predictions were directional (A′, proportion of correct detections, and mean response times), with a significance level of *p* < .05.

The nonparametric measure of bias (B″) did not differ significantly between groups and mean scores were both positive in value indicating a similar conservative bias in both groups towards responding “possible” on the task. The overall measure of sensitivity (A′) showed that ASD participants were significantly less able to discriminate between possible and impossible figures than control participants (although with a small effect size of .23). Group differences on the mean proportion of correct judgments of possible figures and impossible figures, using one-tailed Mann-Whitney tests, did not reach statistical significance.

Wilcoxon’s Signed Ranks tests showed that participants in both groups took longer to respond to impossible figures than possible figures (all *p* < .02). There was no significant group difference in response time to identify either type of figure.

In the ASD group a strong positive association was found between response time and accuracy for possible figures (Spearman rank correlation, *r*_*s*_ = .44, *p* = .03), but not for impossible figures (*r*_*s*_ = −.10, *p* = .62). Williams’ (1959) equation to test the difference between two non-independent correlations showed these two coefficients were significantly different in the ASD group (*t*_(22)_ = 4.71, *p* < .001). No such speed-accuracy trade off was found in the control group (both *r*_*s*_ < −.21, *p* > .26), and no difference in correlations found between the two figure types.

A strong positive relationship was found between FIQ and A′ in the control group (*r*_*s*_ = .72, *p* < .001). The magnitude of the correlation between FIQ and A′ was lower in the ASD group (*r*_*s*_ = .21, *p* = .02), but did not differ significantly from the control group. No association was found between A′ and age in either group (both *r*_*s*_ < .21, *p* > .31).

### Association Between Measures of Global Integration

Correlations between the two measures were run to test the hypothesis that they measure the same underlying construct of global integration. Sensitivity to impossibility was related to the ability to identify objects from fragments across both groups (*r* = −.23, *p* = .05, one-tailed test), which held after controlling for age (*pr* = −.26, *p* = .03), but not FIQ (*pr* = −.03, *p* = .41). The association did not reach significance when separating by group (ASD *r* = −.18, *p* = .20, control *r* = −.22, *p* = .13). However, controlling for the effects of age made this association significant in the control group (*pr* = −.33, *p* = .04) but not in the ASD group (*pr* = −.20, *p* = .17); while controlling for the effects of FIQ reduced this correlation in both groups (ASD *pr* = .02, *p* = .47, control *pr* = −.12, *p* = .26).

## Discussion

Results from the present study supported in part the predictions from Frith’s original conception of weak central coherence in ASD. Although a trend was found on the Fragmented Picture-Completion Task for individuals with ASD to require more fragments of the image to be displayed for correct identification, they were significantly slower to integrate fragments of information than age- and IQ-matched controls in order to identify the degraded pictures, suggesting reduced global integration. Poor integrative processing was also partially demonstrated by ASD participants’ lower sensitivity to global geometrical impossibility compared to controls on the Im/possible Figures Task, albeit with a small effect size. Contrary to predictions, the ASD group were not significantly slower to identify the geometric possibility of figures.

The finding that individuals with ASD required more time to perceive the global form on the Fragmented Picture-Completion Task is in keeping with the conclusion from a recent meta-analysis of local–global research in ASD by Van der Hallen et al. (2015); that the most robust finding is relatively slow global processing. Wang et al. (2007) for example, found that individuals with ASD show their best global performance when stimuli are presented for long exposure times, in contrast to TD individuals where superior global performance is shown for short exposure times. Olu-Lafe et al. (2014) also found a task requiring mental integration took significantly longer for individuals with ASD compared to controls.

Previous studies have shown that the perception of impossibility is indicated by longer looking times to impossible compared to possible forms when asked to draw the figure (Deregowski 1969; Young and Deregowski 1981). A comparable result was found in the present study; participants in both groups took longer to judge impossible figures than possible figures. This suggests that the incongruity of an impossible figure is not an immediate emergent property, but perhaps perceived through a process of systematic integration or serial search. In contrast, the global coherence of possible figures might be hypothesised to have something of a “pop out” effect for typical viewers. It is interesting, therefore, that the present ASD group (unlike the TD controls) showed a significantly stronger speed-accuracy trade-off when judging possible than impossible figures. This may indicate that individuals with ASD were performing an exhaustive serial search on possible figures, with the implication that global coherence is less immediately perceived in individuals with ASD compared to controls. However, the ASD and TD groups were similar in the time taken to decide whether a figure was possible or impossible, which does not fit the speculation that global form showed ‘pop-out’ for the TD but not ASD group. Future studies designed specifically to test “pop-out” in the perception of im/possibility in individuals with ASD would be of interest.

An association was found between visual integration ability and FIQ in the ASD group on the Fragmented Picture-Completion Task, which was not apparent in the control group, suggesting that cohering fragmented information into a perceptual whole is more effortful, or dependent on general processing resources, in the ASD group than in the controls. This may suggest that individuals with ASD have a difficulty integrating information into a meaningful whole and find it more effortful to do so, in keeping with recent reports (e.g., Evers et al. 2014; Olu-Lafe et al. 2014; Scheurich et al. 2010). Given the strong correlation between FIQ and global integration in the ASD group but not the control group, it is of interest to know whether group differences are restricted to, or are more pronounced, in low IQ samples. Further research with larger sample sizes to represent the spectrum of abilities in ASD will be able to address this question further.

There was weak evidence to suggest that both measures tap global integration: the ability to identify images from fragments correlated with the ability to discriminate impossible from possible geometric forms, although this did not reach significance when controlling for FIQ. Indeed, previous literature suggests that such measures would assess discrete constructs. Milne and Szczerbinski (2009), for example, included the Gestalt Completion Task (Ekstrom et al. 1976) and the Impossible-Possible Figures Test (Schacter et al. 1990) in their study of the convergent validity of 14 tasks designed to measure local/global perceptual style. They found no correlation between the two measures of visual integration (*r* = .07, unchanged by controlling for FIQ and choice RT) in their sample of 90 TD adults. Exploratory factor analysis suggested the two tasks also fell onto distinct factors: Impossible Figures loaded strongly onto a *Perceptual Integration* factor, while Gestalt Completion loaded onto a *Cognitive Flexibility* factor. The authors suggest that gestalt closure tasks require the ability to “draw disparate information into a coherent whole” while determining global impossibility requires the ability to “integrate contiguous elements within a single stimulus” (Milne and Szczerbinski 2009; p. 5).

Although the two measures of visual integration in the current study may not tap the same underlying process, the findings provide evidence of reduced global processing in ASD somewhat independently from enhanced local processing. Successful performance on both the Fragmented Picture-Completion Task and the Im/possible Figures Task required the ability to cohere parts into a whole, and this appeared to be hard for the ASD participants. Superior local processing does not provide an obvious alternative explanation for the present group differences. The original notion of wCC as a reduction or disinclination for global processing in the presence of enhanced local processing (Frith 1989) therefore appears to be supported by the present results, which are not easily explained by Enhanced Perceptual Functioning or superior Systemising theories.

Key questions for future research concern the nature of global processing in ASD: Is there an overall slowing of global processing? Does the perception of global form have less immediacy? Is there a natural tendency to local processing in ASD that can only be overcome by effortful global processing? What is the role of cognitive flexibility and other executive functions in local/global processing balance in ASD?

The developmental interplay between putative local processing superiority and reduced global processing also remains to be examined. We do not know whether an early bias towards local details results, developmentally, in a reduced tendency to process global form, or vice versa. For example, difficulty with visual disengagement in infants who later develop autism (Elsabbagh et al. 2013) might be explored in terms of unusual local versus global processing style. Having tasks that tap global processing independent from local bias is a useful first step towards such longitudinal investigations.

Finally, differences in global and local processing in ASD have recently been explained within a Bayesian framework, in several distinct theories (see Pellicano and Burr 2012 and associated commentaries). For example, Van de Cruys et al. (2014) have argued that reduced global processing bias would be one consequence of prediction errors being assigned a uniform and inflexibly high weight in ASD:

…while a familiar representation may not pop-up automatically when a related stimulus appears, top-down activation of holistic, Gestalt-like templates and global processing are often still possible, but as a conscious strategy, when task instructions require it and enough time is available. For individuals with ASD, it is not the default, automatic processing mode. (p. 656)

Such accounts bring us closer to a mechanistic understanding of global processing differences in ASD, ultimately mapable onto neural differences in short and long-range connectivity or inhibitory/excitatory balance (Zikopoulos and Barbas 2013).

## Appendix

Possible and impossible stimuli used in the Im/possible Figures Task.

## Footnotes

1 The values of A′ range from 0 to 1, with a value of 1 indicating perfect discrimination and 0.5 indicating chance performance (an A′ of 0.0 would indicate perfectly inaccurate performance). A′ = 0.5 + [(H − FA)(1 + H − FA)]/[4(H)(1 − FA)] if the hit rate (H) is greater than the false alarm rate (FA), or A′ = 0.5 − [(FA − H)(1 + FA − H)]/[4(FA)(1 − H)] otherwise (Grier 1971).

2 As described by Donaldson (1992), the nonparametric model using the B″ measure of bias appears to be as or more robust than other more widely used signal detection models (i.e., B′, Grier 1971). The values of B″ range from –1.0 to +1.0, with 0.0 indicating no bias, positive values indicating conservative bias (i.e., greater proportion of omissions over false alarms) and negative values indicating a liberal bias (i.e., greater proportion of false alarms over omissions). B″ = [(1 H)(1 FA) − (H)(FA)]/[(1 H)(1 FA) + (H)(FA)].
